# Composite Oxidation Mechanism of Cu/Cu Contact Pairs During Current-Carrying Rolling in O_2_-N_2_-H_2_O Vapor Mixture

**DOI:** 10.3390/ma18245693

**Published:** 2025-12-18

**Authors:** Jianhua Cheng, Fei Li, Yuhang Li, Haihong Wu, Bohan Li, Chenfei Song, Zhibin Fu, Yongzhen Zhang

**Affiliations:** 1 National United Engineering Laboratory for Advanced Bearing Tribology, Henan University of Science and Technology, Luoyang 471023, China230320020269@stu.haust.edu.cn (Y.L.); 18300633747@163.com (B.L.);; 2 National Key Laboratory of Aerospace Mechanism, Shanghai Institute of Aerospace System Engineering, Shanghai 201108, China; feilee88@163.com (F.L.); fuzhibin0903@sina.com (Z.F.); 3 Shanghai Aerospace Equipment Manufacturer Co., Ltd., Shanghai 201100, China

**Keywords:** current-carrying tribology, composite oxidation, humid gas mixture, conductive atomic force microscopy

## Abstract

**Highlights:**

**What are the main findings?**

- Composite oxidation mechanism.
- The ways in which surface oxidation affects the friction performance of current-carrying components.

**What are the implications of the main findings?**

- These results provided an in-depth understanding of the oxidation mechanisms of friction pairs in complex atmospheric environments.

**Abstract:**

Oxidation is a critical factor contributing to material wear and the degradation of conductive performance during current-carrying tribological processes. The present study investigated the composite oxidation mechanisms that occurred during current-carrying rolling in mixed atmospheres containing O_2_ and H_2_O vapor. The results obtained in a dry N_2_/O_2_ mixture, humid N_2_, and humid N_2_/O_2_ mixture indicated that the oxidation mechanisms on current-carrying rolling surfaces involved thermal oxidation, tribo-oxidation, and anodic oxidation. XPS analysis confirmed that the primary oxidation product was CuO. Conductive atomic force microscopy (C-AFM) revealed that surface oxidation caused a significant reduction in conductive α-spots, leading to an increase in contact resistance. Contact resistance exhibited a quasi-linear relationship with the surface CuO content. Contact angle measurements and adhesion tests showed that the enhanced hydrophilicity of the oxidized surface and the resulting high adhesion contributed to an increase in the macroscopic friction coefficient. In humid N_2_/O_2_ with 50% relative humidity (RH), the friction coefficient rapidly exceeded 0.8 when the O_2_ content surpassed 25%. Wear morphology analysis demonstrated that this abrupt increase in the friction coefficient induced fatigue wear on the surface. Overall, the present study elucidated the composite oxidation mechanisms during current-carrying rolling and clarified the pathways through which oxidation affected current-carrying tribological performance. These findings may contribute to improved failure analysis and the safe, reliable operation of electrical contact pairs.

## 1. Introduction

Current-carrying tribo-pairs are widely used in advanced engineering applications such as space solar arrays, radar systems, medical CT scanners, and new-energy vehicles. They serve as critical pathways for power and signal transmission between moving and stationary components [1,2,3]. Current-carrying tribo-pairs often operate under complex service conditions. These harsh environments can significantly degrade their current-carrying tribological performance, potentially causing catastrophic system failure [4,5,6].

In purely mechanical friction, H_2_O and O_2_ in the environment are recognized as major factors influencing tribological performance. Kanao Fukuda et al. [7] have investigated the effects of trace H_2_O and O_2_ on the sliding behavior of pure iron in a H_2_ environment. Their results have shown that the formation of oxides and complex reaction products on the friction surface significantly affects friction and wear performance. Abou Gharam et al. [8] have examined the tribochemical behavior and wear properties of non-hydrogenated diamond-like carbon (DLC) coatings under different O_2_ and humidity environments. Their findings have indicated that in O_2_-rich environments, the synergistic effect of O_2_ and H_2_O promotes the oxidation of carbon atoms on the DLC surface, leading to the formation of O_2_-containing functional groups. This surface modification improves the lubricity of the DLC coating. Cheng et al. [9] have reported that the Ti6Al4V alloy exhibits higher wear volumes in reducing environments, whereas in a moist, oxygen-rich atmosphere (air), the formation of a dense TiO_2_ oxide film on the surface provides a protective effect. The findings from purely mechanical friction have demonstrated that both H_2_O and O_2_ play essential roles in oxidative wear and that surface oxidation alters the tribological properties of materials. However, once an electrical current is introduced into the friction process, surface oxidation becomes far more complex.

Sun et al. [10] have observed that under dripping conditions, the current-carrying rolling surface exhibits both corrosive and fatigue wear, which differs from the damage mechanisms observed under dry conditions. In Wu’s study on Cu-C current-carrying sliding pairs [11], high humidity has been found to cause severe oxidation of Cu, resulting in highly hydrophilic surfaces. Li et al. [12] have reported that under simultaneous humidity and electrical current conditions, the current-carrying tribo-pair undergoes pronounced electrochemical oxidation, thereby degrading its current-carrying tribological performance. Luo et al. [13] have employed conductive atomic force microscopy (C-AFM) and utilized the oxidative effects of humid air to perform nanofabrication experiments based on anodic oxidation lithography. Through precise control of local anodic oxidation, they have successfully fabricated programmable 3D nanostructures. These studies have been conducted in open atmospheric environments, and the results reflect the combined effects of multiple oxidation processes. However, limited research has focused on the individual effects of water and oxygen on oxidation behavior during current-carrying friction.

In the present study, the oxidation mechanism of Cu/Cu contact pairs during current-carrying rolling was investigated in various atmospheric environments, including a dry N_2_/O_2_ mixture, humid N_2_, and a humid N_2_/O_2_ mixture. Based on the surface composition analysis results, the mechanisms of thermal oxidation, friction oxidation, and anodic oxidation were discussed separately, and the roles of H_2_O and O_2_ in surface oxidation were clarified. Based on hydrophilicity and adhesion force tests on the worn surface, the pathways through which oxidation affects friction were analyzed. Using C-AFM imaging, the effect of oxidation on contact conductivity was visually demonstrated. These results provided an in-depth understanding of the oxidation mechanisms of friction pairs in complex atmospheric environments.

## 2. Experimental Details

### 2.1. Test Method of Current-Carrying Rolling Contact

**Figure 1:**
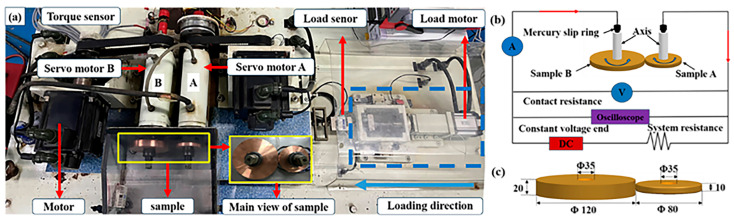
Schematic of the experimental setup: (**a**) FTM-CF100 rolling current-carrying tribotester, (**b**) schematic diagram of the testing principle, and (**c**) sample configuration.

As shown in Figure 1, the experiments were conducted using an FTM-CF100 rolling current-carrying tribometer (Nanjing Bio-inspired Intelligent Technology Co., Ltd., Nanjing, China) [14]. The testing machine was composed of two independently rotating shafts, both supported by insulated bearings. Shaft B was fixed to the equipment base, while Shaft A was mounted on a movable platform. The rolling samples were installed at the top ends of the shafts and were driven to rotate by servo motors. The load motor drove Shaft A toward Shaft B, reducing the distance between the samples until they came into contact. The movement stopped when the load reached a preset value. The load force was measured by a load sensor, and throughout the test, the load was maintained constant through closed-loop control. The rotation power from Servo Motor B was transmitted through a torque sensor to a tension belt, which drove the rotation of sample B. Prior to each test, the torque sensor was calibrated to zero to ensure that the measured torque was generated solely by sample friction. By setting the rotational speeds of Shafts A and B, the linear velocity of the samples was controlled. In the present study, the linear velocities of the two samples were identical.

When the samples were brought into stable contact and reached the preset rotational speed, the DC power supply was connected to the rolling contact pairs. As shown in Figure 1b, the current flowed through the mercury slip rings, the core of Shaft A, Sample A, Sample B, and the core of Shaft B, returning to the mercury slip rings and forming a complete conductive circuit.

During the testing process, the friction torque was acquired using the torque sensor. The friction force (f) was calculated from the ratio of the friction torque (T) to the radius of sample B (L), as given in Equation (1). The friction coefficient (μ) was calculated from the ratio of the friction force (f) to the applied load (Q), according to Equation (2). The voltage between the mercury slip rings (U) and the circuit current (I) were recorded in real time. The circuit resistance was obtained using Ohm’s law. By deducting the static resistance (R_e_) of the slip rings, conductors, shafts, and samples, the contact resistance (R_0_) between the samples was derived (Equation (3)). Re was 20 mΩ, as measured with the micro-ohmmeter (Uni-Trend Technology, UT630, Dongguan, China).

f = T/L(1)

μ = f/Q(2)

R_0_ = U/I − R_e_ (3)

### 2.2. Test Sample

The rolling friction pairs were fabricated from pure Cu (T_2_, GB/T2059-2008), and the geometric configuration is shown in Figure 1c. Sample A had a disk-like shape with a thickness of 10 mm, a radius of 40 mm, and an outer edge curvature radius of 100 mm. Sample B was a disk with a thickness of 20 mm and a radius of 60 mm. Prior to testing, all specimen surfaces were polished using SiC abrasive papers from 800 to 2000 grit. The samples were then ultrasonically cleaned in ethanol for 10 min and dried under a stream of inert gas.

The samples were enclosed in a chamber with an adjustable atmosphere. To investigate the oxidation mechanisms of current-carrying friction surfaces, different oxygen-containing atmospheres were designed, in Table 1. The dry N_2_/O_2_ atmosphere was generated by blending high-purity N_2_ and high-purity O_2_, with the mixing ratio controlled by flowmeters (Weiliang Industrial Control Technology Co., Ltd., QP-2, Guangzhou, China). Humid N_2_ and humid N_2_/O_2_ mixtures were prepared using the saturated salt solution method. Dry gas was pumped into a specific solution, and the humidity of the gas in the bubbles was equilibrated to a stable value. The bubbles then rose to the liquid surface and burst, releasing the humidified gas [15]. The relative humidity was monitored using a humidity sensor (China Resources Microelectronics Technology Co., Ltd., AR847, Shanghai, China).

### 2.3. Test Conditions and Parameters

The test conditions are summarized in Table 2. The rotation speed was set to low-speed slip ring application scenarios, such as radar and pitch motors [18]. The linear velocity of both Sample A and Sample B was 0.025 m/s, with no slip-to-roll ratio. Based on the mechanical properties of Cu (yield strength, σy = 244 MPa, Poisson’s ratio, ν = 0.3, and elastic modulus, E = 110 GPa), the critical contact pressure for yielding of Cu was calculated as 393 MPa according to contact mechanics [19]. The contact pressure produced by the 40 N load in the present study was 240 MPa, indicating that the samples were in a state of elastic contact during the test. The current was set to 1.5 A, which corresponded to the current transmitted by a single-circuit slip ring. During testing, the data acquisition frequency was fixed at 50 Hz, the test time is 100 min. The test data was collected after the break-in period and stabilization. Each test condition was repeated three times to ensure data reliability.

### 2.4. Analysis of Worn Surfaces

After testing, the specimens were sectioned along the radial direction by wire electrical discharge machining (EDM, DK7735, Wei Han CNC, Suzhou, China) for subsequent surface analysis. To investigate the wear mechanisms of the rolling contact pairs, the morphology of the wear tracks was examined using a scanning electron microscope (SEM, ZEISS Sigma 300, Jena, Germany). To analyze the oxidation mechanisms, the elemental composition and chemical states of the contact surfaces were characterized by X-ray photoelectron spectroscopy (XPS, Kratos Axis Supra, Manchester, UK). To elucidate the influence of oxidation on current-carrying tribological performance, the conductive properties (bias: 0.125 V) of the wear surface were characterized, and the adhesive force (bias: 0.16 V) was measured in force curve mode using an atomic force microscope (AFM, Shimadzu SPM-9700HT, Kyoto, Japan). The surface contact angle (CA, Theta Lite, Shenzhen, China) was measured to evaluate the changes in adhesive force.

## 3. Results

### 3.1. Current-Carrying Tribological Performance of Cu/Cu Pairs in Mixed Atmosphere

**Figure 2:**
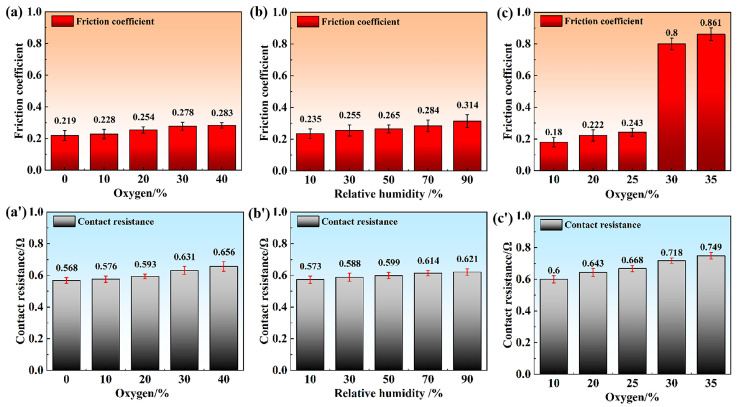
Dependence of (**a**–**c**) Friction coefficient and (**a’**–**c’**) Contact resistance.in different atmospheric conditions: (**a**) dry N_2_/O_2_ mixture, (**b**) humid N_2_ and (**c**) humid N_2_/O_2_ mixture.

Figure 2a illustrates the variation in the friction coefficient in a dry N_2_/O_2_ mixture. As the O_2_ concentration increased from 10 to 40%, the average friction coefficient was measured as 0.219, 0.228, 0.254, 0.278, and 0.283, respectively. Figure 2(a’) presents the corresponding average contact resistance values of 0.568, 0.576, 0.593, 0.631, and 0.656 Ω. These results indicated that increasing O_2_ content had a detrimental effect on both the tribological behavior and electrical conductivity.

Figure 2b shows the change in the friction coefficient in humid N_2_. As the relative humidity increased sequentially from 10 to 90%, the average friction coefficient was measured as 0.235, 0.255, 0.265, 0.284, and 0.314, respectively. Figure 2(b’) presents the corresponding average contact resistance values of 0.573, 0.588, 0.599, 0.614, and 0.621 Ω. These results clearly show that increasing humidity exacerbated the degradation of both tribological and electrical performance.

Figure 2c shows the change in the friction coefficient in a 50% RH humid N_2_/O_2_ mixture. As the O_2_ content increased from 10 to 35%, the average friction coefficient was measured as 0.18, 0.22, 0.243, 0.8, and 0.861, respectively, A distinct abrupt increase in the average friction coefficient occurred when the O_2_ content exceeded 25%. Figure 2(c’) presents the corresponding average contact resistance values of 0.600, 0.643, 0.668, 0.718, and 0.749 Ω. Therefore, in the 50% RH humid N_2_/O_2_ mixture, the increase in O_2_ content had an adverse effect on both tribological and electrical performance.

### 3.2. Surface Characterization

To analyze the wear mechanisms, the morphology and composition of the worn surfaces under different atmospheres were analyzed in detail. To reveal the surface oxidation mechanisms, the author used EDS, XPS, and Raman spectroscopy for surface composition analysis, ultimately selecting XPS as the characterization method (see Supplementary Materials).

**Figure 3:**
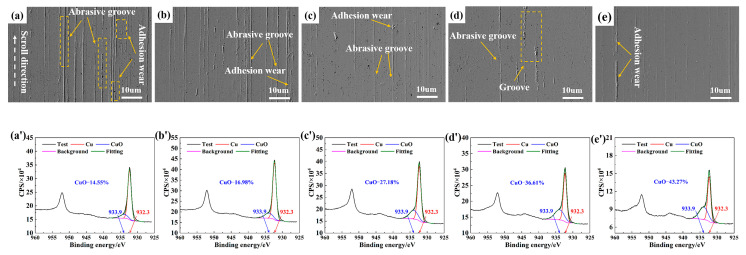
(**a**–**e**) SEM and (**a’**–**e’**) XPS analyses of worn surfaces in dry N_2_/O_2_ mixture with different O_2_ concentrations: (**a**,**a’**) 100% N_2_ (0% O_2_), (**b**,**b**’) 10% O_2_, (**c**,**c’**) 20% O_2_, (**d**,**d’**) 30% O_2_, and (**e**,**e’**) 40% O_2_.

Figure 3a–e illustrate the wear morphology of the sample surfaces after testing in a dry N_2_/O_2_ mixture with different O_2_ concentrations. The observed surfaces were primarily characterized by minor adhesive wear (material transfer was found in the contact area, and irregular tearing marks, dents, and scratches appeared on the surface) and abrasive wear (abrasive grooves and cut marks appear on the surface of the material). As the O_2_ content increased from 10 to 40%, the abrasive wear features on the frictional surfaces gradually decreased.

Figure 3(a’–e’) present the XPS results showing the surface oxidation states of the samples under the corresponding test conditions. Peak deconvolution analysis revealed two characteristic peaks. The peak at a binding energy of 932.3 eV represented metallic Cu (Cu^0^), while the peak at 933.9 eV corresponded to Cu^2+^ species. As the O_2_ content increased from 10% to 40%, the CuO content on the sample surfaces was measured as 14.55, 16.98, 27.18, 36.61, and 43.27%, respectively.

**Figure 4:**
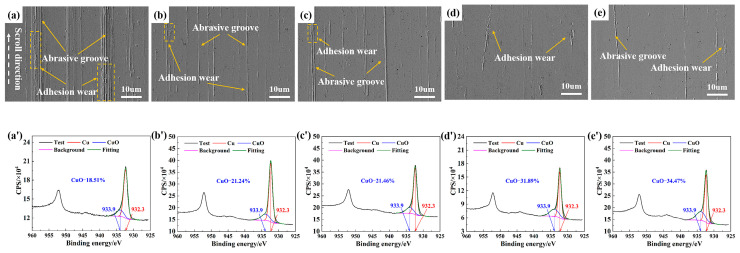
(**a**–**e**) SEM and (**a’**–**e’**) XPS analysis of worn surfaces in humid N_2_ with different humidity levels: (**a**,**a’**) 10% RH, (**b**,**b’**) 30% RH, (**c**,**c’**) 50% RH, (**d**,**d’**) 70% RH, and (**e**,**e’**) 90% RH.

Figure 4 shows the wear morphology and oxidation levels of the sample surfaces in humid N_2_ under different humidity conditions. Similar phenomena were observed, where the surface damage was mainly characterized by minor adhesive and abrasive wear. Simultaneously, the XPS results showed that as the humidity increased from 10 to 90% RH, the CuO content on the sample surfaces rose from 18.51 to 34.47%, With the increased oxidation level, a corresponding reduction in both adhesive and abrasive wear was observed on the sample surfaces.

**Figure 5:**
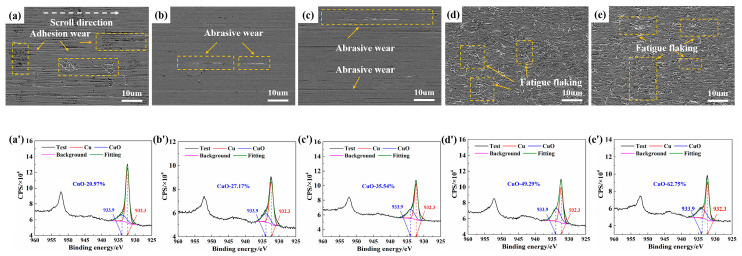
(**a**–**e**) SEM and (**a’**–**e’**) XPS analysis of worn surfaces in humid N_2_/O_2_ mixture with different O_2_ concentrations: (**a**,**a’**) 10% O_2_, (**b**,**b’**) 20% O_2_, (**c**,**c’**) 25% O_2_, (**d**,**d’**) 30% O_2_, and (**e**,**e’**) 35% O_2_.

Figure 5 illustrates the wear morphology and oxidation levels of the sample surfaces in a humid N_2_/O_2_ mixture with different O_2_ concentrations. In humid atmospheres with low to moderate O_2_ content (Figure 5a–c), adhesive and abrasive wear were still present, whereas in high-O_2_ humid conditions (Figure 5d,e), a new wear mechanism emerged, predominantly characterized by fatigue wear (cracks initiate and propagate in the sub-surface or surface of the material). As previously reported, an abrupt transition in the friction coefficient occurred under these conditions. The elevated friction coefficient led to intensified shear stress at the contact interface, thereby promoting fatigue wear [20].

XPS analysis (Figure 5(a’–e’)) revealed that when the O_2_ content in humid atmospheres increased from 10 to 35%, the oxidation level of the friction surfaces rose significantly from 16.31 to 62.75%. This oxidation level was substantially higher than that observed in dry N_2_/O_2_ mixture or humid N_2_ conditions. These results indicated that the synergistic effect of H_2_O and O_2_ significantly accelerated surface oxidation.

In a review on advances in current-carrying tribology, Li et al. [1]. noted that the coefficient of friction during current-carrying sliding is influenced by multiple factors. These include adhesive effects between metallic surfaces, increased hydrophilicity and adhesion due to oxidation, as well as electric-field-induced capillary actions. Meanwhile, variations in contact resistance are closely related to the content of surface oxides. Therefore, to investigate the mechanisms behind the performance evolution during current-carrying friction, the oxidation processes under electrical sliding are analyzed in detail in the following discussion. Moreover, conductive atomic force microscopy (C-AFM) and adhesion force measurements were performed to elucidate how oxidation influence the current-carrying tribological behavior.

## 4. Discussion

As revealed by the above microscopic characterization, O_2_, H_2_O vapor, and their mixture could each induce oxidation on the current-carrying friction surface. Oxidation had a detrimental influence on both the friction coefficient and the contact resistance. A sudden surge in the friction coefficient triggered fatigue wear on the rolling surface. The following section provides a detailed discussion of the composite oxidation mechanism and its influence on current-carrying tribology.

### 4.1. Composite Oxidation Mechanism

**Figure 6:**
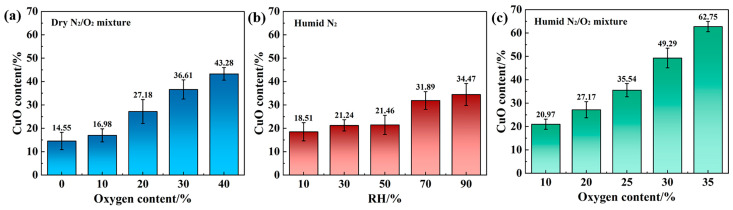
CuO content on worn surfaces under different atmospheric environments: (**a**) dry N_2_/O_2_ mixture, (**b**) humid N_2_, and (**c**) 50% humid N_2_/O_2_ mixture.

The results in Figure 6a indicated that Cu underwent oxidation in a dry N_2_/O_2_ mixed gas, which was classified as solid-state oxidation [21]. The oxygen required for oxide formation in this atmosphere was derived solely from O_2_. The combined effects of Joule heating and frictional heating increased the localized temperature at the contact area, thereby accelerating the oxidation reaction kinetics. This thermal activation promoted the adsorption of O_2_ molecules on the Cu surface and their solid-state diffusion, which consequently accelerated the formation of the oxide layer [22]. Mechanical rolling continuously exposed fresh metal surfaces and reduced the diffusion barrier, thereby allowing thermal oxidation to proceed further. The thermal oxidation reaction pathway primarily followed the sequence: Cu → Cu_2_O → CuO [23]. However, due to the thermodynamic instability of Cu(I) (prone to disproportionation), it decomposes into Cu(0) and Cu(II) [24]. Therefore, it is difficult to detect the presence of Cu(I) on the worn surfaces of the samples.

The results in Figure 6b demonstrated that H_2_O vapor also initiated oxidation. The O_2_ required for the oxide formation in this atmosphere was derived solely from H_2_O vapor. The oxidation mechanism of the Cu disk during current-carrying rolling in humid N_2_ was governed by the coupled effects of tribo-oxidation and anodic oxidation. During rolling, the adsorption of water may lead to the formation of a meniscus between contact asperities, and the hydroxylation of water on the Cu surface generated hydrophilic terminal groups in the contact region. Subsequently, the input of frictional energy facilitated a dehydration reaction between hydroxyl-terminated groups, resulting in the formation of Cu–O–Cu bridging bonds [25,26]. During subsequent sliding, the Cu-O-Cu bonds absorbed frictional energy during the shear process, forming high-energy chemical bonds. These bonds were prone to hydrolysis upon interaction with adsorbed H_2_O molecules, leading to the formation of CuO [27,28]. A more detailed explanation of the mechanisms of friction oxidation is provided in Ref. [29].

Meanwhile, anodic oxidation driven by electric current also proceeded. The H_2_O vapor acted as both an oxidizing agent and an electrolyte. Electrons participated in the cathodic reduction reaction at the cathode (the conductive contact zone of the tribo-pair) [30]:

2H_2_O + 2e^−^ → H_2_ + 2OH^−^ (4)

The oxidation reaction of Cu occurred at the anode:

Cu + 2OH^-^ → Cu(OH)_2_ + 2e^−^ (5)

Joule heating and frictional heating synergistically promoted the thermal decomposition and dehydration reactions of Cu(OH)_2_ [31]:

Cu(OH)_2_ → CuO + H_2_O (6)

Under open atmospheric conditions, both H_2_O vapor and O_2_ participated in current-carrying tribo-oxidation. Previous studies have extensively investigated the effects of air with varying humidity levels on oxidation. They have found that the degree of oxidation on the current-carrying wear surface increases with rising humidity. In the present study, while maintaining a constant humidity level (50% RH), the O_2_ content in the N_2_/O_2_ mixture was varied. The results in Figure 6c indicated that the extent of oxidation on the current-carrying wear surface increased with increasing O_2_ content. Figure 6 demonstrated that the most severe oxidation occurred on the contact surface of the current-carrying tribo-pair in a humid O_2_ environment.

### 4.2. Pathways of Oxidation Effects on Current-Carrying Tribological Performance

The results of the present study demonstrated that oxidation modified the composition and damage behavior of the rolling surface, thereby influencing current-carrying tribological contact. While numerous researchers have examined the impact of oxidation on contact resistance and the coefficient of friction, a direct and visual representation of this relationship has not yet been reported. Based on the microscopic observations obtained from AFM, this section aimed to clarify the pathways through which oxidation affected macroscopic current-carrying tribological properties.

**Figure 7:**
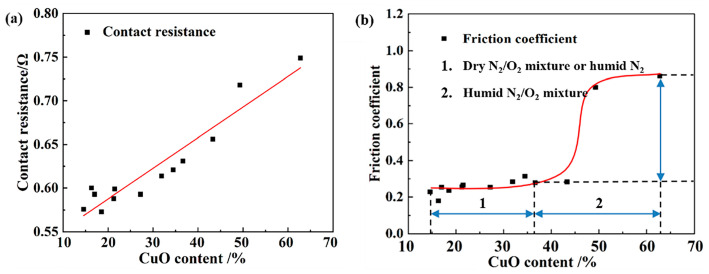
Effect of oxidation level on current-carrying tribological performance. (**a**) contact resistance; (**b**) friction coefficient.

**Figure 8:**
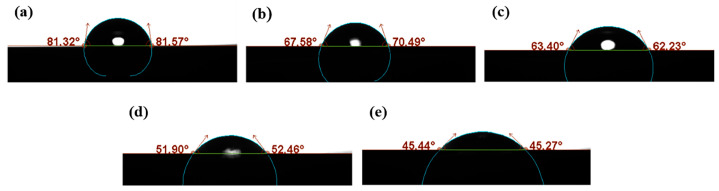
Contact angle measurements on wear surfaces under different oxidation levels: (**a**) pure Cu, (**b**) 27.17% CuO, (**c**) 36.61% CuO, (**d**) 43.28% CuO, and (**e**) 49.29% CuO.

**Figure 9:**
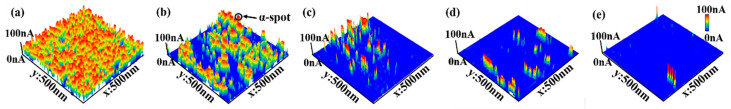
Conductive AFM images of the wear surface under different oxidation levels: (**a**) pure Cu, (**b**) 27.17% CuO, (**c**) 36.61% CuO, (**d**) 43.28% CuO, and (**e**) 49.29% CuO. The constant bias voltage applied to the samples was −0.125 V.

**Figure 10:**
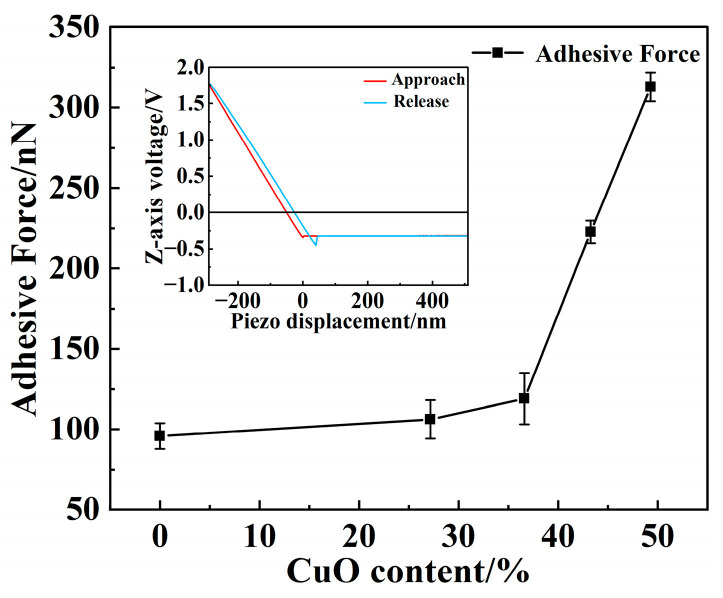
Adhesive force measured by AFM on wear surfaces under different oxidation levels.

The effect of oxidation level on current-carrying tribological performance is summarized in Figure 7. A quasi-linear relationship existed between the contact resistance and surface oxidation level (Figure 8a). While previous studies have primarily attributed this phenomenon to the poor electric conductivity of CuO [19,32], the present study provided a visualized explanation based on C-AFM results (Figure 9). As is well known, contact conductivity depends on the properties and distribution of conductive α-spots [33]. Figure 10 illustrates the surface conductivity distribution obtained under C-AFM mode, showing that most of the pure Cu surface exhibited conductive behavior. The conductive regions were distributed in a spot-like pattern over the friction surface. As the degree of surface oxidation increased, these conductive α-spots decreased significantly. Therefore, oxidation increased the contact resistance by reducing the number of conductive α-spots.

As shown in Figure 7b, a nonlinear relationship was observed between the friction coefficient and the oxidation level. Under low oxidation levels or dry conditions (humid N_2_ or dry N_2_/O_2_), the friction coefficient increased slowly. In contrast, under high oxidation levels or humid conditions (synergy between H_2_O and O_2_), a sharp upward trend was observed. The results indicated that the combined action of humidity and oxidation led to a rapid increase in the current-carrying friction coefficient during rolling. This variation was likely associated with the adhesion forces of hydrophilic surfaces [20,34]. This section aimed to clarify the influence of oxidation on the friction coefficient from the perspectives of hydrophilicity and adhesion forces.

The surface hydrophilicity of the samples shown in Figure 8 was evaluated using contact angle measurements, and the results are presented in Figure 10. The oxidation levels were 0% (pure Cu under ideal conditions), 27.17, 36.61, 43.28, and 49.29%, and the corresponding contact angles were 81.32, 67.58, 63.40, 51.90, and 45.44°. These results indicated that CuO exhibited a higher water adsorption capability than pure Cu. The increased hydrophilicity of the oxidized surface leads to an enhanced adhesive force in the micro-contact regions, resulting in an elevation of the friction coefficient. Figure 10 illustrates the adhesive forces measured using the force-distance curve mode of AFM in open air at 50% RH. As the CuO content increased from 0 to 49.29%, the surface adhesive force rose progressively from 95.8 to 312.9 nN, with a pronounced surge observed when the CuO content exceeded 40%. When hydrophilic surfaces came into contact in a humid atmosphere, a capillary meniscus could form around the contact asperities, resulting in significant adhesion in the contact area. Upon separation of the rolling surfaces, additional work was required to overcome this adhesion, leading to an increase in the friction coefficient. The adhesion test results were consistent with the macroscopic friction coefficient results.

In summary, to investigate the oxidation mechanisms of the current-carrying rolling surface, the present study conducted a series of tests under different atmospheric conditions. The results obtained in dry N_2_/O_2_ mixtures indicated the presence of thermal oxidation on the current-carrying rolling surface. The tests performed in humid N_2_ revealed that tribo-oxidation and anodic oxidation also occurred. The most severe surface oxidation observed in the humid N_2_/O_2_ atmosphere suggested that these three oxidation mechanisms collectively contributed to the overall surface oxidation. Furthermore, from the perspectives of microscopic conductive α-spots and adhesive forces, the pathways through which oxidation influenced current-carrying tribological performance were elucidated. It can be concluded that in an open atmospheric environment, the combined effects of thermal oxidation, tribo-oxidation, and anodic oxidation result in a higher degree of oxidation on current-carrying friction surfaces compared to conventional mechanical friction. Oxidation not only increases contact resistance but also elevates the friction coefficient through enhanced surface adhesion. The findings of the present study could contribute to a deeper understanding of the surface oxidation mechanisms and performance degradation processes in current-carrying rolling systems.

## 5. Conclusions

The present study investigated the composite oxidation mechanisms on the current-carrying rolling surface and their effects on the conductive and tribological performance of Cu/Cu pairs. Based on experiments performed under different atmospheric conditions, distinct oxidation mechanisms were identified. Using analytical techniques such as SEM, XPS, and AFM, the pathways through which oxidation influenced current-carrying rolling performance were elucidated. The conclusions were as follows:

1. Based on the experimental results obtained under dry N_2_/O_2_ mixtures, humid N_2_, and humid N_2_/O_2_ atmospheres, thermal oxidation, tribo-oxidation, and anodic oxidation collectively contributed to the composite oxidation of Cu/Cu contact pairs during current-carrying rolling. The highest degree of oxidation occurred under humid N_2_/O_2_, with XPS analysis confirming CuO as the primary surface oxidation product.
2. C-AFM results revealed that surface oxidation caused a significant reduction in conductive α-spots, thereby increasing the macroscopic contact resistance. Contact resistance exhibited a quasi-linear relationship with the surface CuO content.
3. The oxidized surface exhibited enhanced hydrophilicity and greater adhesion, resulting in an elevated friction coefficient. The increase in the friction coefficient subsequently promoted the initiation of surface fatigue wear.

## Figures and Tables

**Table 1:** Atmosphere conditions and parameters.

Atmosphere	Preparation Method	Ingredients/Content
Dry N_2_/O_2_ mixture	The preparation was carried out by mixing dry gases, with O_2_ content controlled through flow rate regulation.	O_2_ content: 0%, 10%, 20%, 30%, 40%/vol.%
humid N_2_	Dry gas was introduced into the saturated electrolyte solution to achieve different humidity levels [16].	humidity level: 10%, 30%, 50%, 70%, 90%.
50% humidified N_2_/O_2_ mixture	Dry N_2_/O_2_ mixture gas was introduced into the saturated electrolyte solution to achieve 50% humidity [17].	O_2_ content:10%, 20%, 25%, 30%, 35%/vol.%

**Table 2:** Test conditions and parameters.

Parameters	Value
Rotational speed of sample A	6 rpm
Rotational speed of sample B	4 rpm
Slip-to-roll ratio	0%
Linear speed	0.025 m/s
Normal contact load	40 N
Contact pressure	240 MPa
Current intensity	1.5 A
Test time	100 min

## Data Availability

The original contributions presented in this study are included in the article and Supplementary Materials. Further inquiries can be directed to the corresponding author.

## Supplementary Materials

The following supporting information can be downloaded at: https://www.mdpi.com/article/10.3390/ma18245693/s1, Figure S1: EDS of the sample surface area in humid N_2_: (a) 10%RH, (b) 30% RH.; Figure S2: XPS analysis of sample surface under 50% RH humid N_2_/O_2_ mixture (35% O_2_) conditions; Figure S3: Raman tests under different atmospheric conditions. References [20,35,36,37] are cited in the Supplementary Materials.
