# Supplementary material for: Composite Oxidation Mechanism of Cu/Cu Contact Pairs During Current-Carrying Rolling in O2-N2-H2O Vapor Mixture
Source: Materials (Basel). 2025 Dec 18;18(24):5693. doi: 10.3390/ma18245693 (PMC12734869; doi:10.3390/ma18245693)
Supplement: Supplementary file 1 [file materials-18-05693-s001.zip › materials-3995046-supplementary.pdf]

Composite oxidation mechanism of Cu/Cu contact pairs during current-carrying rolling in O_2-N_2-H_2O vapor mixture

Jianhua Cheng [1, 2], Fei Li [2], Yuhang Li [1], Haihong Wu [3], Bohan Li [1], Chenfei Song [1, *], Zhibin Fu [2], Yongzhen Zhang [1]

[1]National United Engineering Laboratory for Advanced Bearing Tribology, Henan University of Science and Technology, Luoyang, 471023, PR China

[2]National Key Laboratory of Aerospace Mechanism, Shanghai Institute of Aerospace System Engineering, Shanghai, 201108, PR China

[3]Shanghai Aerospace Equipments Manufacturer Co., Ltd, Shanghai, 201100, PR China

*Corresponding author: cfsong@haust.edu.cn

Supplementary Analysis

In order to effectively carry out the mechanistic analysis in the process of tribo-chemistry, in addition to the SEM and Cu^{2+} XPS analyses presented in the main text, we also conducted EDS analysis, XPS analysis of OH^- and O^{2-}, as well as Raman tests. The related results help to better understand the content of the main text.

Figure S1 shows the EDX analysis results of the worn areas of the samples in pure N_2 environments with different humidity levels. Quantitative analysis of the spectra reveals that the O:Cu ratio on the sample surfaces is similar, with 0.116 at 10% RH and 0.115 at 30% RH. We found that the EDX results under different conditions did not show significant differences. This may be related to factors such as surface roughness and material depth of the samples, which could lead to large errors in EDX measurements. In addition, the relatively low atomic number of oxygen makes it difficult to detect in EDX [1].

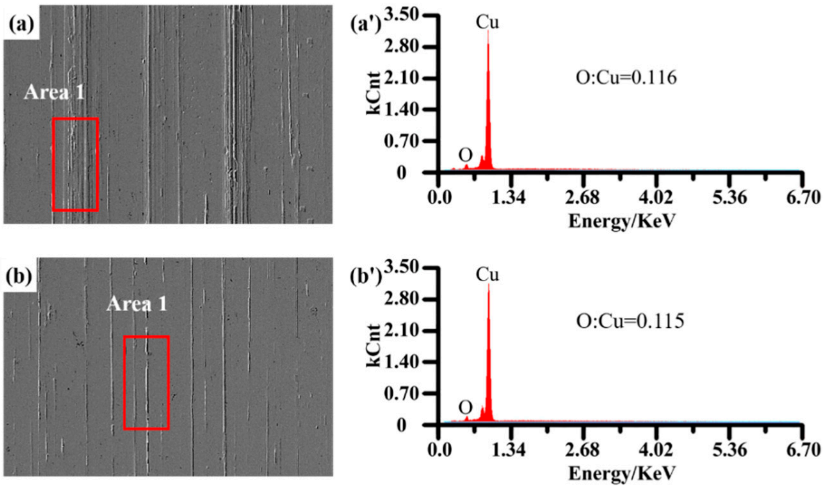

Figure S1 EDS of the sample surface area in humid N_2: (a) 10%RH, (b) 30% RH.

Figure S2 shows the XPS test results of the sample surface under N_2/O_2 (35% oxygen content) conditions at 50% relative humidity. Data fitting analysis revealed three characteristic peaks, with the peak at a binding energy of 932.3 eV representing metallic copper (Cu^0), the peak at 933.9 eV corresponding to Cu^{2+} species, and the peak at 529.6 eV corresponding to O^{2-} species. Additionally, only a very small amount of OH^- was detected at a peak position of 531.5 eV on this sample surface, with a relatively weak signal. This may be due to water adsorption during friction under humid conditions; it could also be an intermediate product of a tribo-chemical reaction [2].

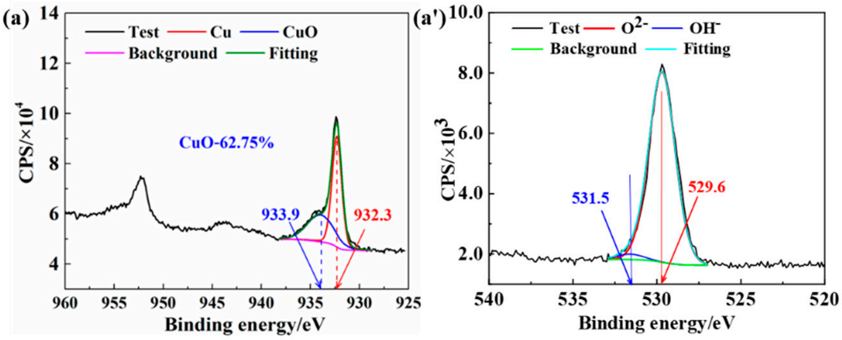

Figure S2 XPS analysis of sample surface under 50% RH humid N_2/O_2 mixture (35% O_2) conditions

Figure S3 shows the Raman spectra of samples prepared in dry N_2/O_2 mixtures, humid N_2, and 50% humidified N_2/O_2 mixtures. A weak $Cu_2(OH)_2CO_3$ signal was observed at 1400 cm^{-1}; a peak corresponding to adsorbed H_2O was found at 1600 cm^{-1}; C-H bonds were detected at around 2900 cm^{-1}. These peaks are related to the adsorption of the samples in open air, possibly due to minor carbon contamination and water adsorption occurring during sample unpacking and transfer [3, 4].

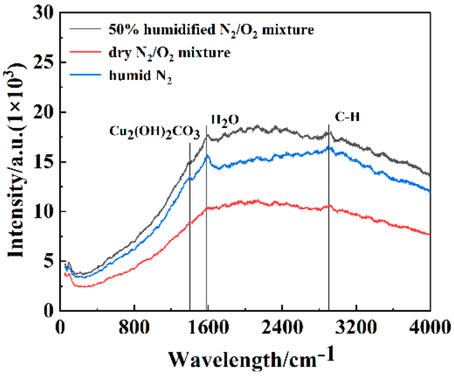

Figure S3 Raman tests under different atmospheric conditions

References

[1] Newbury* E, Ritchie M. Is Scanning Electron Microscopy/Energy Dispersive X-ray Spectrometry (SEM/EDS) Quantitative? Scanning. 2013, 35(3): 141-68.

[2] Sun Y, Song C, Zhang Y, et al. Oxidation on the current-carrying rolling surface and its subsequent impact on the damage of Cu contact pairs in O_2/N_2 mixture. Materials Letters. 2021, 288(129349.

[3] Deng Y, Handoko D, Du Y, et al. In Situ Raman Spectroscopy of Copper and Copper Oxide Surfaces during Electrochemical Oxygen Evolution Reaction: Identification of CuIII Oxides as

Catalytically Active Species. ACS Catalysis. 2016, 6(4): 2473-81.

[4] Chen S, Li H, Jiang W, et al. MOF Encapsulating N-Heterocyclic Carbene-Ligated Copper Single-Atom Site Catalyst towards Efficient Methane Electrosynthesis. Angewandte Chemie International Edition. 2021, 61(4).
